# Improvement in radiation techniques for locally advanced cervical cancer during the last two decades

**DOI:** 10.1136/ijgc-2022-004230

**Published:** 2023-04-11

**Authors:** Satoru Sagae, Takafumi Toita, Motoki Matsuura, Manabu Saito, Takuma Matsuda, Nanaka Sato, Ayumi Shimizu, Toshiaki Endo, Miho Fujii, David K Gaffney, William Small

**Affiliations:** 1 Women’s Medical Center, Tokeidai Memorial Hospital, Sapporo, Hokkaido, Japan; 2 Radiation Therapy Center, Okinawa Chubu Hospital, Uruma, Okinawa, Japan; 3 Department of Obstetrics and Gynecology, Sapporo Medical University, Sapporo, Hokkaido, Japan; 4 Department of Radiation Oncology, Huntsman Cancer Institute, University of Utah, Salt Lake City, Utah, USA; 5 Department of Radiation Oncology, Loyola University Chicago, Stritch School of Medicine, Cardinal Bernardin Cancer Center, Maywood, Illinois, USA

**Keywords:** cervical cancer, radiotherapy, conformal, radiotherapy, image-guided, radiotherapy, intensity-modulated, radiology, interventional

## Abstract

Since the National Cancer Institute (NCI) alert of concurrent chemoradiotherapy, radiotherapy has been changed from external beam radiotherapy plus brachytherapy to platinum-based concurrent chemoradiotherapy. Therefore, concurrent chemoradiotherapy plus brachytherapy has become a standard treatment for locally advanced cervical cancer. Simultaneously, definitive radiotherapy has been changed gradually from external beam radiotherapy plus low-dose-rate intracavitary brachytherapy to external beam radiotherapy plus high-dose-rate intracavitary brachytherapy. Cervix cancer is uncommon in developed countries; hence, international collaborations have been critical in large-scale clinical trials. The Cervical Cancer Research Network (CCRN), created from the Gynecologic Cancer InterGroup (GCIG), has investigated various concurrent chemotherapy regimens and sequential methods of radiation and chemotherapy. Most recently, many clinical trials of combining immune checkpoint inhibitors with radiotherapy have been ongoing for sequential or concurrent settings. During the last decade, the method of standard radiation therapy has changed from three-dimensional conformal radiation therapy to intensity-modulated radiation therapy for external beam radiotherapy and from two-dimensional to three-dimensional image-guided approaches for brachytherapy. Recent improvements include stereotactic ablative body radiotherapy and MRI-guided linear accelerator (MRI-LINAC) using adaptive radiotherapy. Here we review the current progress of radiation therapy during the last two decades.

## Introduction

For the definitive treatment of locally advanced cervical cancer, definitive radiation therapy plus brachytherapy with concurrent cisplatin is the standard of care. After multiple clinical trials, radiation therapy and concurrent platinum-based chemotherapy demonstrated an overall survival benefit compared with radiation therapy alone.^1^ However, while concurrent chemoradiotherapy has improved clinical outcomes, it is not without associated normal tissue toxicity. In particular gastrointestinal toxicity concerns are worsened, primarily when extended-field irradiation treats para-aortic metastasis. While considering the benefit of radiation therapy, the past two decades have witnessed an expansion in radiation therapy options for the concurrent and sequential management of cervical cancer. These ideas include enhancing the radiation effect with concurrent chemotherapy, standardization of radiation techniques, and further advancements in radiation modalities, especially with international collaboration. Therefore, to promote the distribution and usefulness of radiation treatment globally one should review the following issues: (1) Gynecologic Cancer InterGroup (GCIG) and Cervical Cancer Research Network (CCRN) – these activities have aided the standardization of radiation therapy; (2) improving external beam radiation therapy plus brachytherapy, with rapidly distributing intensity-modulated radiation therapy, and three-dimensional image-guided adaptive brachytherapy; and (3) new modalities of radiation techniques, such as stereotactic ablative body radiotherapy boost or MRI-guided linear accelerator (MRI-LINAC) using adaptive radiotherapy. Herein, we explore the clinical benefit of these novel strategies currently being tested in clinical trials in cervical cancer and their optimal use to improve radiation therapy.

## International Collaboration to Perform Clinical Trials

In 1999, the National Cancer Institute (NCI) of the United States published a clinical alert indicating a survival benefit for adding platinum-based chemotherapy to radiotherapy in International Federation of Gynecology and Obstetrics (FIGO) stages IB2–IVA.^1^ Meta-analysis has confirmed the survival advantage of chemoradiotherapy over radiotherapy alone.^2^ Also, some studies have documented the rapid incorporation of platinum-based chemoradiotherapy as standard treatment within a short period after the NCI 1999 clinical alert. Concerning the benefit of concurrent chemoradiotherapy plus brachytherapy, a meta-analysis of individual patient data from 18 randomized chemoradiotherapy trials for cervical cancer in 2008 showed a 6% improvement in 5-year survival with chemoradiotherapy.^3^ In Japan, the Japanese Gynecologic Oncology Group (JGOG) assessed the feasibility and acute toxicity of concurrent chemoradiotherapy plus brachytherapy with cisplatin in JGOG1066.^4^ They showed concurrent chemoradiotherapy with high-dose-rate intracavitary brachytherapy and standard weekly delivery of cisplatin was feasible with acceptable toxicity in Japanese patients with cervical cancer and also revealed the excellent efficacy and toxicity of concurrent chemoradiotherapy plus brachytherapy. Furthermore, Surveillance, Epidemiology, and End Results (SEER) analysis^5^ demonstrated overall survival improvement for cervical cancer patients treated in the era of concurrent chemoradiotherapy plus brachytherapy. Therefore, concurrent chemoradiotherapy plus brachytherapy as a standard of care for locally advanced cervical cancer was supported by population-level evidence in this study. Moreover, recently a randomized controlled trial of concurrent chemoradiotherapy plus brachytherapy from India showed significantly better disease-free survival and overall survival than radiation alone in women with stage IIIB cervical cancer.^6^

## Trials of Chemoradiotherapy plus Brachytherapy

Clinical trials of concurrent chemoradiotherapy plus brachytherapy have been conducted using platinum compounds, such as weekly versus triweekly (TACO), and combinations of platinum with other drugs such as gemcitabine/cisplatin.^7^ Gemcitabine plus cisplatin during concurrent chemoradiotherapy, followed by brachytherapy and adjuvant gemcitabine/cisplatin chemotherapy, improved survival outcomes but was associated with much higher toxicity than the standard treatment. The OUTBACK trial^8^ randomized patients with locally advanced cervical cancer to standard concurrent chemoradiotherapy plus brachytherapy with weekly cisplatin versus concurrent chemoradiotherapy plus brachytherapy with four cycles of adjuvant carboplatin and paclitaxel. With a median follow-up of 60 months, 5-year overall survival was 72% adjuvant carboplatin and paclitaxel versus 71% control, and 5-year progression-free survival was 63% versus 61%. Grade >3 adverse events occurred in 81% of the adjuvant carboplatin and paclitaxel group compared with 62% of the control. It was concluded that adjuvant chemotherapy after standard concurrent chemoradiotherapy plus brachytherapy does not improve overall survival or progression-free survival and increases toxicity.

While concurrent chemoradiotherapy with a platinum agent and brachytherapy has been the standard of care for multimodality therapy in cervical cancer, novel strategies with targeted agents and immunotherapy have been actively tested in clinical trials in the definitive and metastatic setting. Radiation can activate the immune system when combined with immunotherapy, resulting in a synergistic response. However, multiple controversies remain regarding the treatment for locally advanced cervical cancer, including the optimal immunotherapy combination with radiotherapy and durvalumab^9^ or pembrolizumab,^10^ and the radiotherapy dose when given in the primary or metastatic setting. In the CALLA trial,^9^ durvalumab in combination with and following concurrent chemoradiotherapy plus intracavitary±interstitial brachytherapy did not significantly improve progression-free survival in patients with high-risk locally advanced cervical cancer compared with concurrent chemoradiotherapy plus brachytherapy alone (Table 1).

**Table 1:** Current ongoing clinical trials of chemoradiotherapy for locally advanced cervical cancer

Trial name	Phase	Interventions	Radiation details	Primary endpoint	Current status
OUTBACK NCT01414608	Ⅲ 2012–2018	Control: CCRT Investigational: CCRT+adjuvant CT (paclitaxel+carboplatin) Enrollment: 926 participants	EBRT: 3D-CRT BT: 2D-ICBT, 3D-CT/MRI-based IGBT (IC/IS BT is not allowed)	OS	ASCO 2021 Plenary^8^ CCRT+adjuvant CT vs CCRT OS at 5 years: 72% vs 71% (HR 0.91) PFS at 5 years: 63% vs 61% (HR 0.87) NS in OS and PFS
TACO NCT01561586	Ⅲ 2012–2023	Control: CCRT Investigational: CCRT (triweekly CDDP) Enrollment: 374 participants	EBRT: 2D-/3D-CRT BT: 2D-ICBT, 3D-CT/MRI-based IGBT (IC/IS BT is not allowed)	OS	Active, not recruiting
INTERLACE NCT01566240	Ⅲ 2012–2026	Control: CCRT Investigational: NAC (paclitaxel+carboplatin) →CCRT Enrollment: 500 participants	EBRT: 3D-CRT, IMRT BT: 2D-ICBT, 3D-CT/MRI-based IGBT (IC/IS BT if indicated)	OS	Active, not recruiting
CALLA NCT03830866	Ⅲ 2019–2023	Control: CCRT Investigational: CCRT+durvalumab (Concurrent→adjuvant) Enrollment: 770 participants	EBRT: 3D-CRT or IMRT BT: 2D-ICBT, 3D-CT/MRI-based IGBT (IC/IS BT if indicated)	PFS	IGCS 2022 Plenary^9^ Durvalumab+CCRT vs placebo+CCRT HR 0.84 (95% CI 0.65–1.08); p=0.174 N.S. in PFS
MK-3475-A18/ KEYNOTE-A18/ ENGOT-cx11/ GOG-3047 NT04221945	Ⅲ 2020–2024	Control: CCRT Investigational: CCRT+pembrolizumab (Concurrent→adjuvant) Enrollment: 980 participants	EBRT: 3D-CRT or IMRT BT: 2D-ICBT, 3D-CT/MRI-based IGBT (IC/IS BT if indicated)	PFS at 38 months OS at 46 months	*J Clin Oncol* 2020^10^ Active, not recruiting
NRG-GY006 NCT02466971	Ⅲ 2016–2023	Control: CCRT Investigational: CCRT+triapine Enrollment: 450 participants	EBRT: 3D-CRT or IMRT BT: 2D-ICBT, 3D-CT/MRI-based IGBT (IC/IS BT if indicated)	OS	Active, not recruiting
EMBRACE Ⅱ NCT03617133	Ⅱ 2016–2031	Control: CCRT (Historical: Retro-/EMBRACE) Investigational: CCRT Enrollment: 1000 participants	EBRT: IMRT BT: 3D-MRI-based IGBT (IC/IS BT if indicated) Increased use of IC/IS BT, reduction of vaginal source loading, protocol for target and OAR contouring, EBRT dose prescription and reporting, adaptation of EBRT nodal elective CTV according to risk of nodal and systemic recurrence, use of IMRT and IGRT for EBRT delivery, reduction of overall treatment time	Local control for 5 years, nodal control for 5 years, systemic control for 5 years, OS for 5 years, overall morbidity for 5 years, EORTC QoL for 5 years	*Int J Radiat Oncol Biol Phy*s 2019^41^ Active, not recruiting

ASCO, American Society of Clinical Oncology; BT, brachytherapy; CCRT, concurrent chemoradiation therapy plus brachytherapy; CI, confidence interval; CR, complete response; CRT, conformal radiotherapy; CT, computed tomography; CT, chemotherapy; CTV, clinical target volume; 2D, two-dimensional; 3D, three-dimensional; EBRT, external beam radiotherapy; EORTC, European Organization for Research and Treatment of Cancer; HR, hazard ratio; HR-QoL, health-related quality of life; ICBT, intracavitary brachytherapy; IC/IS, intracavitary/interstitial; IGBT, image-guided brachytherapy; IGCS, International Gynecologic Cancer Society; IMRT, intensity-modulated radiation therapy; MRI, magnetic resonance imaging; NAC, neoadjuvant chemotherapy; NS, no statistically significant improvement; OAR, organs at risk; PFS, progression-free survival; PR, partial response; QoL, quality of life.

## International Obstacles and Approaches

The United States National Clinical Trials Network has recently expanded beyond North America to include Canadian research bases, and international collaboration is anticipated for several United States-based research bases. In addition, the current NRG Oncology group has expanded to other international sites in Asia and Europe. Specific to gynecologic malignancies, the GCIG was formalized in 1997 as a collaborative network of international research groups performing clinical trials in gynecologic cancers. A recent global survey of oncologists regarding the obstacles to international clinical trials cited lack of funds as the most critical factor in low- and middle-income countries. In addition, regulatory procedures were ranked as the next most crucial impediment to clinical research in this setting. The GCIG surveyed the practice patterns of radiotherapy in cervical cancer among member groups to describe the therapeutic practice of treating cervical cancer.^11^ For the treatment of advanced cervical cancer, pelvic external beam doses and total doses to point A was 47 Gy and 79.1 Gy, respectively. More than 80% of groups used concurrent chemoradiotherapy plus brachytherapy, using weekly cisplatin. Among member groups of the GCIG, radiotherapy practices were similar in terms of both doses and the use of chemotherapy. These surveys belong in the age of two-dimensional intracavitary brachytherapy, while in the current era of three-dimensional intracavitary brachytherapy, a new standardization and monitoring of these distributions are required.

Furthermore, under the current rapid advancement of radiation techniques, including intensity-modulated radiation therapy as external radiation, three-dimensional image-guided (MRI-based) brachytherapy, the American Society for Radiation Oncology (ASTRO),^12^ NRG Oncology,^13^ and the international study group on MRI-based brachytherapy in locally advanced cervical cancer (EMBRACE)^14^ propose recommendations of target delineation/organs at risk contouring and standardization of dose constraints. In the real world, penetration of techniques and an indication of possibilities of new modalities for radiation techniques will be necessary to monitor and survey the technical advantages.^15^ To successfully perform global clinical trials of cervical cancer, including concurrent chemoradiotherapy plus brachytherapy and immuno-oncology, novel standardization of radiotherapy will be resolutely required in the future.^16^

## The Cervix Cancer Research Network (CCRN)

The GCIG established the Cervix Cancer Research Network (CCRN) after its Cervical Cancer State of the Science Meeting held in Manchester in 2009.^17^ The vision of the CCRN was to accredit individual sites to have a sufficiently high quality of treatment and follow-up and adequately trained staff and resourced infrastructure to contribute to international trials according to good clinical practice standards. The challenge facing the CCRN is that of funding. The International Gynecologic Cancer Society (IGCS), recognizing the potential of the CCRN, has generously donated an unrestricted grant to the CCRN, which the CCRN has matched. The CCRN was designed to provide clinical trials to women in low- and middle-resource settings.

In November 2014, a cervical cancer brainstorming meeting in Melbourne, Australia entitled “Advances and Concepts in Cervical Cancer Trials: A Road Map for the Future” was held during the GCIG meeting, and discussed the future direction of cervical cancer trials, including surgery, chemotherapy, radiation therapy, and molecular issues.^18^ As a result, the GCIG had grown to include 28 member groups, and there was significant worldwide interest in the CCRN. At that time, 17 different CCRN site visits were performed, and there were 10 approved CCRN sites. In addition, CCRN currently has three multinational publicly funded clinical trials using radiotherapy open for enrollment in low-, middle-, and high-income countries.

The triweekly cisplatin-based chemoradiation in locally advanced cervical cancer (TACO) trial [NCT01561586], which investigators developed from the Korean Gynecologic Oncology Group (KGOG) and the Thai Cooperative Group, compared weekly cisplatin cancer therapy to every-3-week chemotherapy for locally advanced cervical cancer. In addition, the concurrent chemoradiotherapy plus brachytherapy with carboplatin and paclitaxel in patients with locally advanced cervical cancer (OUTBACK) trial [NCT01414608]^8^ was performed globally, as noted earlier. The induction chemotherapy plus chemoradiation plus brachytherapy as first-line treatment for locally advanced cervical cancer (INTERLACE) trial [NCT01566240] is headed by the National Cancer Research Institute from the United Kingdom. This trial evaluates the administration of neoadjuvant chemotherapy before definitive chemoradiation therapy plus intracavitary±interstitial brachytherapy (Table 1).

In 2015, a CCRN report mentioned the global outreach effort.^19^ In January 2016, in Bangkok, Thailand, a CCRN educational workshop was held to distribute the advance of radiotherapy for cervical cancer in Asia, with participating members from surrounding countries.^20^ Sixty-two participants attended from 16 different countries. This symposium evaluated progress, promoted new clinical trials for the CCRN, and educated on brachytherapy in treating cervical cancer. In addition, the CCRN held its second international educational symposium in Mexico City with 90 participants from 15 Latin American countries in January 2017^21^; the third and fourth annual CCRN symposia were held in Romania^22^ and South Africa, respectively. In addition, the CCRN has opted to hold meetings in regions of the world with a high rate of cervix cancer. The CCRN symposium for Vietnam in 2020 was planned but canceled due to the COVID pandemic, with plans for the seminar to be accomplished in 2023.^23^

## Advances in External Beam Radiation Therapy (EBRT) and Brachytherapy (ICBT)

### Advances in Brachytherapy

In the 1990s and 2000s, brachytherapy was usually performed using an intracavitary approach with intrauterine tandem and vaginal colpostats. However, depending on the patient and tumor anatomy, in patients with an intact cervix, the vaginal component of brachytherapy may be delivered using ovoids, ring, or cylinder brachytherapy (combined with the intrauterine tandem). Initially, low-dose-rate intracavitary brachytherapy was standard. However, since the American Brachytherapy Society (ABS) recommended high-dose-rate intracavitary brachytherapy in 2000,^24^ high-dose-rate has been recognized to have similar efficacy and toxicity. In addition, high-dose-rate has apparent advantages compared with low-dose-rate, including decreased dose to staff by using a remote afterloading system and capability in the outpatient setting. In 2012, the GCIG surveyed brachytherapy practice patterns^25^ to determine current practice patterns concerning gynecologic high-dose-rate intracavitary brachytherapy among GCIG members in Japan/Korea, Australia/New Zealand, Europe, and North America. Among 72 responses, 61 respondents (85%) utilized a high-dose-rate. The fractionation patterns were varied, but the overall mean dose administered for cervical cancer was similar in Australia/New Zealand, Europe, and the United States. Members in Japan administered a significantly lower external beam dose and higher brachytherapy dose to the cervix. Furthermore, similar to the JGOG1066 study,^4^ high-dose-rate intracavitary brachytherapy was planned with classical two-dimensional images and point A dose description. Then the results suggested that high-dose-rate intracavitary brachytherapy would improve pelvic control using modern three-dimensional image-guided brachytherapy, which could deliver appropriate doses to the entire volume of the locally advanced tumors without increasing the surrounding organs.

### Image-Guided Brachytherapy

Although treatments were historically planned using two-dimensional films and the point-based Manchester system in the United States and Europe, technical advances have led to increased use of CT or MRI for three-dimensional volumetric planning.^26^ MRI provides a much better definition of tissues, allowing adaptive planning as the tumor regresses with each delivered fraction. In 2005, the Groupe Europeen de Curietherapie and the European Society for Radiotherapy and Oncology (GEC-ESTRO) published guidelines for optimal MRI-guided brachytherapy planning target volumes.^27^ The total prescription dose is the combined external beam and brachytherapy biologically effective dose delivered in 2 Gy fractions (EQD2). Image-guided brachytherapy improves local control and reduces normal tissue toxicity. In 2012, the French STIC trial,^28^ the first prospective, non-randomized trial to compare two-dimensional versus three-dimensional brachytherapy in treating locally advanced cervical cancer, showed that three-dimensional brachytherapy was feasible and safe in routine practice. It improved local control with half the toxicity observed with two-dimensional dosimetry. In 2017, the American Brachytherapy Task Group^29^ reported a pooled analysis of clinical outcomes for high-dose-rate brachytherapy for cervical cancer, which showed an improvement in outcomes with the use of image-guided brachytherapy compared with traditional point A dose prescriptions, and demonstrated that high-dose-rate brachytherapy is a safe, effective modality when combined with image-guided brachytherapy. As reported in 2019, the international RetroEMBRACE study improved local control and reduced late toxicity for women with large tumors treated with image-guided brachytherapy relative to historical controls.^30^ Early results of the prospective, multi-institutional EMBRACE study have identified improved dose–volume thresholds for urinary and rectal toxicity in women with locally advanced cervical cancer receiving MRI-guided brachytherapy. For patients with prominent irregularly shaped tumors, sufficient dose coverage is difficult to be achieved during whole dose constraint keeping organs at risk with the standard intracavitary brachytherapy. The therapeutic advantage of hybrid intracavitary and interstitial brachytherapy has been reported.^31^ In the EMBRACE I study, concurrent chemoradiotherapy with MRI-based image-guided brachytherapy was effective and stable for long-term local control across all stages of locally advanced cervical cancer.^32^ The currently open EMBRACE II study [NCT03617133] further optimizes the therapeutic ratio using the latest external beam radiation therapy and brachytherapy techniques using new dedicated applicators combined with conventional intracavitary and interstitial needles (Table 1).

### Intensity-Modulated Radiation Therapy (IMRT)

Radiation therapy is the primary treatment for patients with locally advanced cervical cancer. However, traditional radiation therapy has adverse effects, including cystitis, proctitis, enteritis, small bowel obstruction, and fistulas. Especially with external beam radiation therapy, conventional three-dimensional conformal radiation therapy showed a particular incidence of severe side effects on the small bowel and bone marrow. Conversely, intensity-modulated radiation therapy treats areas of interest while limiting the dose to normal tissues and may be a way to reduce toxicity from radiation therapy and possibly increase the dose and improve outcome. For example, intensity-modulated radiation therapy for irradiation of the para-aortic nodal chain is also likely to decrease the risk of toxicities compared with two-dimensional/three-dimensional radiation therapy, while allowing dose escalation to intact positive nodes, especially for patients receiving concurrent chemotherapy.^33^ However, no data show that intensity-modulated radiation therapy improves disease-specific survival or overall survival over two-dimensional/three-dimensional techniques.

An up-to-date contouring atlas and guidelines for imaging during intensity-modulated radiation therapy are needed. In addition, continuous upfront monitoring for both contouring and planning of patients^34^ and managing internal volume changes and motion is required, possibly with image-guided radiotherapy.^35^ The dose-calculation algorithms for intensity-modulated radiation therapy shape the dose around target structures with multiple converging beams or arcs. This approach makes it possible to reduce the dose delivered to the normal tissues that surrounde the target structures. However, intensity-modulated radiation therapy must address patient positioning, contouring, and set-up reproducibility to avoid errors with these highly conformal approaches. Given the technical capacity to reduce treatment volumes to spare normal tissue, a reproducible treatment planning set-up and the careful use of relevant diagnostic imaging and thorough physical examination are critical for optimal treatment planning.

Indeed, CT simulation aims to replicate the positioning that will probably be encountered during daily treatment, given the possibility for large targets and organs at risk of movement due to rectum and bladder filling status. Moreover, appropriate internal margins need to be added to the clinical target volume, especially for the primary lesion, to form the internal target volume and subsequently to the planning target volume to keep adequate dose coverage for the clinical target volume. It is also essential to apply image-guided radiotherapy for daily intensity-modulated radiation therapy delivery. Once CT simulation is complete and diagnostic imaging is fused or otherwise carefully examined, the accurate delineation of both target volumes and organs at risk is crucial to optimize the probability of tumor control and minimize toxicity. There are consensus contouring guidelines for both intact and post-operative scenarios to assist with target volume segmentation.^36 37^ With the advent of three-dimensional imaging, target structures should be contoured to ensure radiotherapy field design reflects individual variations in patient anatomy. Target nodal sites (regions) for the definitive treatment of cervical cancer are the common iliac, internal iliac, external iliac, obturator, and presacral lymph nodes. Normal tissues, including the small bowel, bladder, rectum, sigmoid, pelvic bones, and femoral heads, are also contoured. When para-aortic nodes are being treated, limiting the dose to the duodenum, kidney, spinal cord, and liver is appropriate.

In the post-operative setting, more recent evidence suggests that intensity-modulated radiation therapy may decrease toxicity while maintaining excellent oncologic outcomes. In the randomized Radiation Therapy Oncology Group (RTOG) 1203 study,^38^ intensity-modulated radiation therapy significantly reduced gastrointestinal and genitourinary toxicity compared with the standard four-field approach from the patient’s perspective. The PARCER trial^39^ also showed image-guided intensity-modulated radiation therapy with reduced toxicity with no difference in disease outcomes, as well as the most current trial of positron emission tomography-guided bone marrow-sparing intensity-modulated radiation therapy for locally advanced cervical cancer.^40^ Similarly, definitive concurrent chemoradiotherapy with intensity-modulated radiation therapy plus brachytherapy is expected to be a promising strategy for locally advanced cervical cancer. Additionally, intensity-modulated radiation therapy significantly reduced acute gastrointestinal and genitourinary toxicities and chronic genitourinary toxicity in patients with cervical cancer, which is strongly recommended by ASTRO.^12^

### Intensity-Modulated Radiation Therapy plus Image-Guided Brachytherapy

Using image-guided brachytherapy in cervical cancer, the clinical results of these innovations were presented based on the multicenter EMBRACE I and RetroEMBRACE studies with large patient cohorts (n=1416, n=731, respectively).^30 32^ Image-guided brachytherapy for cervical cancer improves pelvic control and survival across all stages. Improving pelvic control is more significant in advanced stages, but progress in survival is similar across stages. The RetroEMBRACE cohort study analyzed the failure patterns to investigate this discrepancy.^30^ Some 731 patients from 12 institutions treated with chemoradiation therapy and MRI- or CT-based image-guided brachytherapy were evaluated. In addition, this study analyzed the pattern of failure at the time of the first relapse. After image-guided brachytherapy, the predominant failure is systemic, whereas the principal failure with conventional brachytherapy is pelvic. As an evolution of practice from EMBRACE-I to -II,^32 41^ the importance of technique, target selection, contouring, dose prescription, and dose-planning in external beam radiation therapy for cervical cancer was emphasized as follows: EMBRACE-I involved 1416 patients with locally advanced cervical cancer treated with chemoradiation, including image-guided brachytherapy during 2008 to 2015. EMBRACE II, which is now enrolled and has accrued 1000 patients, comprises a comprehensive, detailed strategy and accreditation procedure for external beam radiation therapy with target contouring, treatment planning, and image guidance, performing three-dimensional conformal radiation therapy or intensity-modulated radiation therapy as external beam radiation therapy and two-dimensional intracavitary brachytherapy or three-dimensional MRI-based image-guided brachytherapy (intracavitary±interstitial brachytherapy if indicated). External beam radiation therapy planning target volumes (PTVs), treated volumes (V43 Gy), and conformity index (CI; V43 Gy /PTV) will have been evaluated in both studies and compared. A similar report^42^ of intensity-modulated radiation therapy/three-dimensional image-guided brachytherapy for cervical cancer from the United States was associated with improved survival and decreased gastrointestinal and genitourinary toxicity in patients with cervical cancer compared with those who received two-dimensional external beam radiation therapy and brachytherapy.

### Extended-field irradiation and brachytherapy

Another approach is extended-field irradiation and intracavitary brachytherapy with or without chemotherapy for patients with positive para-aortic or high common iliac lymph nodes. The RTOG 0116 trial^43^ was designed to test the toxicity of combined chemotherapy with extended-field irradiation and intracavitary brachytherapy. Patients received extended-field irradiation of 45 Gy (1.8 Gy/fraction) in addition to intracavitary brachytherapy, which estimated 85 Gy low-dose-rate equivalent to the final point A dose. The boosted irradiation was 54 to 59.4 Gy for the positive para-aortic and high common iliac lymph nodes. Cisplatin (40 mg/m^2^) was delivered weekly during external beam radiation therapy and once with brachytherapy. Among 26 eligible patients with para-aortic metastasis or high common iliac involvement, 16 (62%) patients had a complete response for both local and nodal disease. However, the acute and late grade 3/4 toxicity rate was 81% and 40%, respectively. This toxicity might be because of no use of intensity-modulated radiation therapy. In conclusion, extended-field irradiation and intracavitary brachytherapy with cisplatin for para-aortic or highly common iliac node metastasis from cervical cancer were associated with significant acute and late toxicity.

A recent study^44^ in 2018 was performed among eligible patients with locally advanced cervical cancer and documented positive para-aortic lymph nodes, extended-field irradiation, and brachytherapy with concurrent cisplatin 40 mg/m^2^ weekly for 6 weeks. Some 4–6 weeks after completing concurrent chemoradiotherapy, patients were treated with four cycles of paclitaxel 135 mg/m^2^ and escalating doses of carboplatin with area under the curve (AUC) 4 or 5. The therapeutic value of prophylactic extended-field irradiation for patients with a high risk of para-aortic lymph node recurrence is another critical issue. Furthermore, extended-field, intensity-modulated radiotherapy with concurrent chemotherapy was tolerated well, with acceptable toxicities in patients with cervical cancer and para-aortic lymph node metastasis. Moreover, an Indian study^45^ in 2019 was undertaken to report the early toxicity with extended-field, intensity-modulated radiotherapy for cervical cancer in their cohort of patients and determine dose–volume parameters that predict over grade 2 hematological toxicity and diarrhea. Thus, extended-field, intensity-modulated radiotherapy was feasible for cervical cancer patients with para-aortic lymph node involvement and associated with acceptable grade 3 toxicity.

## New Modalities of Radiation Techniques

### Stereotactic Ablative Body Radiotherapy

Currently, controversial issues exist as to whether high-quality external beam radiation therapy can be an alternative to brachytherapy for cervical cancer. Despite these excellent brachytherapy results, the use of brachytherapy is declining in the United States.^46^ Recently, the use of stereotactic ablative body radiotherapy has rapidly increased in clinical practice for various cancers such as lung, kidney, and prostate. As a salvage/palliative option for locoregional and distant recurrences and oligometastatic cervical cancer,^47^ local control and toxicity associated with stereotactic ablative body radiotherapy seem reasonable for most clinical indications during short follow-ups, for example, for some patients ineligible for brachytherapy.^48^ However, before applying this strategy as an alternative to brachytherapy in definitive treatment in clinical practice, further investigation is necessary regarding target volume delineation, the minimum dose required to control tumors, and patterns of internal organ motion (inter-/intra-fractions). In addition, one phase II clinical trial evaluating stereotactic ablative body radiotherapy was closed early due to toxicity, and local control was only 70% at 2 years.^49^ Therefore, stereotactic ablative body radiotherapy should not be used as a routine alternative to brachytherapy, although some clinical guidelines do not recommend it.^34^ The stereotactic ablative body radiotherapy boost for cervical tumors is still investigational, and the available data suggest worse outcomes with non-brachytherapy approaches.^25^ No studies have been published directly comparing stereotactic ablative body radiotherapy with brachytherapy.

### MRI-LINAC Using Adaptive Radiotherapy

Many image-guided radiotherapy techniques have been developed, but motion can be random and difficult to predict before treatment. In addition, MRI-guided treatment planning is more complex for external beam radiation therapy than for brachytherapy, requiring electron density information and a whole-body contour for accurate dose calculations. Nevertheless, many different technical solutions have been developed, including a combined MRI and cobalt radiotherapy unit, an MRI scanner on rails, and a linear accelerator combined with an MRI scanner (MRI-LINAC).^50^ ^50^ Compared with CT, these approaches have potential benefits, including MRI simulation allowing for more accurate and reproducible contouring, improved visualization of the tumor for accurate localized dose escalation, imaging during external beam radiation therapy to enable the management of inter- and intra-fraction variations, and dose–response assessment with multiparametric MRI to guide further treatment. Therefore, with its superior soft-tissue contrast, MR-guided radiation therapy can potentially reduce toxicity and potentiate dose escalation in external beam radiation therapy for cervical cancer.

## Future Directions

Among radiotherapy plus chemotherapy, such as concurrent, adjuvant, and neoadjuvant styles, the control of distant metastasis during or after radiotherapy is crucial, so many trials are ongoing. Unfortunately, the current OUTBACK trial^8^ did not show any advantage of adjuvant chemotherapy with many difficulties in understanding the results, such as patients' characteristics, stage, used drug and dose, the timing between radiotherapy and chemotherapy, and others. The ongoing TACO trial expects to show some evidence of concurrent chemotherapy compared with weekly and triweekly platinum. It might reveal some results with better disease control, side effects, and quality of life during radiation therapy. The INTERLACE trial is also trying to show similar results of radiation control under the setting of neoadjuvant chemotherapy. Therefore, establishing the best way to add chemotherapy to radiotherapy is necessary to maximize disease control and minimize toxicities, so we need to generate more well-designed trials globally that will define the position of chemotherapy in locally advanced cervical cancer treatment (Table 1).

Ongoing studies of concurrent chemoradiotherapy plus brachytherapy in combination with immune checkpoint inhibitors in patients with locally advanced cervical cancer are assessing the sequence of treatments and overall efficacy and safety. For example, recent results from the phase III CALLA trial^9^ showed that durvalumab, in combination with and following concurrent chemoradiotherapy plus brachytherapy, did not significantly improve progression-free survival in patients with locally advanced cervical cancer, with no new or unexpected toxicity. Furthermore, the phase III ENGOT-cx/KEYNOTE-A18 trial^10^ evaluating the combination of pembrolizumab with concurrent chemoradiotherapy plus brachytherapy is ongoing, and the results of this trial are highly anticipated. They will further elucidate whether immunotherapy combined with definitive concurrent chemoradiotherapy plus brachytherapy can improve local control, pelvic control, and survival in patients with locally advanced cervical cancer without significantly increasing toxicities.

EMBRACE II prescribes MRI-guided adaptive brachytherapy with combined intracavitary±interstitial techniques and specific dose–volume constraints for adaptive targets and organs at risk and image-guided external beam radiotherapy for particular targets and techniques (intensity-modulated radiation therapy, image-guided radiation therapy, simultaneously integrated boost for lymph node boosting, and more para-aortic radiotherapy) and concurrent chemoradiotherapy plus brachytherapy. EMBRACE II intends to benchmark excellent local, nodal, distant control and survival rates, morbidity, and quality of life outcomes and prospectively evaluate the evidence derived from the previous RetroEMBRACE^30^ and EMBRACE I^32^ studies. These results will be used as a reference in many centers worldwide and in clinical studies reflecting clinical, biological, and technical parameters of importance for further optimizing the therapeutic ratio for chemoradiotherapy and intracavitary brachytherapy in locally advanced cervical cancer.

## Conclusions

During the last two decades, radiation therapy has rapidly improved from external beam radiation therapy plus low-dose-rate intracavitary brachytherapy to external beam radiation therapy plus high-dose-rate intracavitary brachytherapy. Since the NCI alert concerning concurrent chemoradiotherapy, definitive radiation therapy for locally advanced cervical cancer has changed dramatically from external beam radiation therapy plus brachytherapy alone to external beam radiation therapy with concurrent platinum-based chemotherapy plus brachytherapy. In many countries, intensity-modulated radiation therapy is preferred over three-dimensional conformal radiation therapy for gynecological applications where the bladder, rectum, bowel, and bone marrow are proximal. Various chemotherapeutic regimens have been tested in international clinical trials, and strategies of neoadjuvant chemotherapy before radiation therapy or adjuvant chemotherapy after concurrent chemoradiotherapy plus brachytherapy are ongoing. Moreover, numerous clinical trials of immune checkpoint inhibitors combined with concurrent chemoradiotherapy plus brachytherapy are also ongoing. External beam radiation therapy and brachytherapy have changed from three-dimensional conformal radiation therapy to intensity-modulated radiation therapy and from two-dimensional intracavitary brachytherapy to three-dimensional image-guided brachytherapy. Current investigations include stereotactic ablative body radiotherapy or MRI-LINAC using adaptive radiotherapy; however, there is no firm evidence that these techniques are clinically advantageous. Radiation therapy continues to be the most attractive strategy to treat locally advanced cervical cancer, with high local control rates. However, some recent trials have indicated that the most common site of the first failure is distant; hence, improved systemic therapies are needed.
